# Early prediction of extubation failure in patients with severe pneumonia: a retrospective cohort study

**DOI:** 10.1042/BSR20192435

**Published:** 2020-02-07

**Authors:** He Yu, Jian Luo, Yuenan Ni, Yuehong Hu, Dan Liu, Maoyun Wang, Binmiao Liang, Zongan Liang

**Affiliations:** 1 Department of Critical Care Medicine, West China School of Medicine and West China Hospital, Sichuan University, Chengdu, China; 2 Department of Respiratory and Critical Care Medicine, West China School of Medicine and West China Hospital, Sichuan University, Chengdu, China

**Keywords:** APACHE II score, blood glucose, extubation failure, fentanyl, RBC transfusion, severe pneumonia

## Abstract

Backgroud: Severe pneumonia is one of the most common causes for mechanical ventilation. We aimed to early identify severe pneumonia patients with high risk of extubation failure in order to improve prognosis.

Methods: From April 2014 to December 2015, medical records of intubated patients with severe pneumonia in intensive care unit were retrieved from database. Patients were divided into extubation success and failure groups, and multivariate logistic regressions were performed to identify independent predictors for extubation failure.

Results: A total of 125 eligible patients were included, of which 82 and 43 patients had extubation success and failure, respectively. APACHE II score (odds ratio (OR) 1.141, 95% confident interval (CI) 1.022–1.273, *P* = 0.019, cutoff at 17.5), blood glucose (OR 1.122, 95%CI 1.008–1.249, *P* = 0.035, cutoff at 9.87 mmol/l), dose of fentanyl (OR 3.010, 95%CI 1.100–8.237, *P* = 0.032, cutoff at 1.135 mg/d), and the need for red blood cell (RBC) transfusion (OR 2.774, 95%CI 1.062–7.252, *P* = 0.037) were independent risk factors for extubation failure.

Conclusion: In patients with severe pneumonia, APACHE II score > 17.5, blood glucose > 9.87 mmol/l, fentanyl usage > 1.135 mg/d, and the need for RBC transfusion might be associated with higher risk of extubation failure.

## Introduction

Pneumonia is a common respiratory infectious disease and is one of the top six causes of death [1]. Approximately 10% of hospitalized patients with pneumonia require intubation for invasive mechanical ventilation (IMV) due to respiratory failure, which is termed as severe pneumonia [2,3]. IMV provides essential life support and a treatment window by increasing arterial oxygenation, rebalancing ventilation/perfusion ratio, and decreasing respiratory workload, and eventually improves prognosis and reduces mortality. However, the long-term use of IMV may result in hazardous complications, such as ventilator-associated pneumonia, barotrauma, oxygen toxicity, and increased use of sedatives and cost [4,5]. Furthermore, it has been reported that prolonged weaning was associated with higher hospital mortality [6]. Therefore, early identification of pneumonia patients with risk of extubation failure is of great clinical significance because interventions might be planned and implemented when risk could be predicted and monitored.

Previous studies have found a variety of factors are associated with extubation failure, including: (1) demographics and clinical status, such as age, fluid balance, comorbidity burden, cardiac function, mental status, and severity of illness; (2) laboratory abnormalities, such as hypercapnia, serum albumin, and blood urea nitrogen (BUN); (3) respiratory mechanics, such as maximal inspiratory pressure and diaphragmatic function; and (4) treatment interventions, such as sedatives [5,7–9]. However, these predictors are identified from a cluster of diseases including pneumonia, chronic obstructive pulmonary disease, heart failure, and trauma. Moreover, pneumonia and acute respiratory distress syndrome (ARDS) have been elucidated to be independent predictors for extubation failure [9].

Based on the lack of specific investigations of predictors and risks for extubation failure predictors in patients with severe pneumonia, we retrospectively analyzed the data from intubated patients with severe pneumonia and aimed to identify the independent predictors for extubation failure.

## Methods

From April 2014 to December 2015, medical records of the patients, who were admitted to Respiratory Intensive Care Unit (RICU) of West China Hospital, Sichuan University, were retrieved from the hospital inpatient database system. Study protocol was approved by the Institutional Ethical Committee for Clinical and Biomedical Research of West China Hospital, and all patients or their legal substitute decision makers were consented for publication of in-hospital data. All methods were performed in accordance with the relevant guidelines and regulations released by the Chinese National Institutes of Health and the Clinical Trial Center of West China Hospital.

### Patients

Patients were eligibly included if they were diagnosed of severe pneumonia upon admission and needed IMV via oral intubation or tracheostomy for more than 24 h due to acute respiratory failure. Severe pneumonia was defined if patients met either one of the major criteria (acute respiratory failure requiring IMV and septic shock with need for vasopressors) or at least three minor criteria (respiratory rate ≥ 30 bpm, ratio of partial pressure of arterial oxygen to fraction of inspired oxygen (PaO_2_/FiO_2_) ≤ 250, BUN ≥ 20 mg/dl, white blood cell count < 4 × 10^9^/l, platelet count < 100 × 10^9^/l, body temperature < 36°C, multilobar infiltrates, confusion/disorientation, and hypotension requiring aggressive fluid resuscitation) [2]. The indication for IMV was evaluated based on the arterial blood gas (ABG) analysis indicating refractory hypoxemia with PaO_2_ less than 50 mmHg although on oxygen supplement.

We excluded patients if their pneumonia was secondary to known pulmonary diseases such as acute exacerbation of chronic obstructive pulmonary disease (COPD), asthma, and pulmonary thromboembolism, or if they were in fatal and life-threatening comorbidities including, but not limited to, aggressive carcinoma with unstable clinical conditions and cerebral injury. Patients with immunosuppressive therapy were also excluded in our study.

### Study design

The present study was designed as a retrospective cohort study, in which eligible patients were classified into extubation success and failure groups. Patients’ baseline demographics and clinical characteristics, laboratory measurements, imaging information, and treatment details at the time of ICU admission were compared between these two groups to identify potential independent risk factors for the prediction of extubation failure in patients with severe pneumonia.

### Definition of extubation success and failure

Successful extubation was defined as removal of endotracheal tube for more than 72 h regardless of the need for sequential non-invasive positive pressure ventilation (NPPV); while extubation failure was defined as unavoidable reintubation within 72 h of extubation [10]. Failure of IMV discontinuation was not treated as extubation failure only if the patients failed the extubation afterward. All patients who fell into the extubation failure criteria were mechanically ventilated after the trial of extubation or ventilator discontinuation.

### Ventilation and treatment strategies

In general, mechanical ventilation was initiated with a tidal volume (*V*_T_) of 8–10 ml/kg of predicted body weight and a minimal positive end-expiratory pressure (PEEP), which was titrated with FiO_2_ to target an oxygen saturation of pulse oximetry (SpO_2_) ≥ 90% or PaO_2_ ≥ 60 mmHg using pressure-controlled or volume-controlled assist/control mode [11,12]. However, “lung protective ventilation” and “open-lung approach” were given if patients developed ARDS, in which the patients’ lungs were protected by a lower *V*_T_ of 4–8 ml/kg of predicted body weight with a plateau airway pressure ≤ 30 cm H_2_O and the collapsed alveoli were opened by intermittent recruitment maneuvers and maintained open by a PEEP of 2 cm H_2_O higher than pressures at which an abrupt increment was detected in the upward slope of the pressure–volume curve [11,12]. Respiratory therapists up- or down-regulated the support of ventilation based on the ABG analysis every day.

Empirical antibiotics treatment was administered in accordance with the consensus by the Infectious Diseases Society of America and American Thoracic Society, and reasonable changes were made once a specific pathogen was identified and considered as clinical importance [2]. All patients were sedated with midazolam and (or) propofol on the basis of analgesia with fentanyl to meet a goal of Richmond Agitation-Sedation Scale between -2 and 0 and a target of critical care pain observation tool < 3. Daily interruption by temporarily ceasing the sedative infusions was also implemented to avoid potential delirium, which was defined if Richmond Agitation-Sedation Scale was between −3 and +4 and Confusion Assessment Method for Intensive Care Unit was positive [13,14]. Blood glucose level was measured every 4 h routinely and insulin dosing was commenced when two consecutive blood glucose levels are greater than 10 mmol/l with a target upper blood glucose ≤ 10 mmol/l [15]. Other treatments including but not limited to organ function supports, airway clearance by bedside bronchoscopy, circulation and nutrition maintenance, as well as internal environment homeostasis stabilization were also performed when necessary according to patients’ clinical conditions.

### Extubation procedures

Spontaneous breathing trial (SBT) was conducted by respiratory therapists if the following criteria were met: (1) the causes of mechanical ventilation were relieved or alleviated; (2) PaO_2_/FiO_2_ > 150–200 at FiO_2_ ≤ 0.4–0.5 and PEEP ≤ 5–8 cm H_2_O; (3) stable hemodynamics without need for vasopressors or dopamine/dobutamine <5–10 μg/kg/min; and (4) the capability of spontaneous breathing. [16]. During SBT, a low level of pressure support between 5 and 7 cm H_2_O was used for 30 min, and patients were considered as readiness for extubation if they have no following conditions: (1) agitation, anxiety, depressed mental status, and diaphoresis; (2) evidence of increased accessory muscle activity; (3) PaO_2_ ≤ 50–60 mmHg at FiO_2_ ≥ 0.5 or arterial oxygen saturation (SaO_2_) < 90%; (4) partial pressure of arterial carbon dioxide (PaCO_2_) > 50 mmHg or an increase of PaCO_2_ > 8 mmHg; (5) pH < 7.32 or a decrease of pH ≥ 0.07 pH units; (6) respiratory rate (RR) > 35 breaths per minute (bpm) or increased by ≥ 50%; (7) rapid shallow breathing index (RSBI = ratio of RR to *V*_T_) > 105 bpm; (8) heart rate > 140 beats per minute or increased by ≥20%; (9) systolic blood pressure < 90 mmHg or > 180 mmHg or increased by ≥ 20%; and (10) cardiac arrhythmias [17]. Otherwise, the previous ventilation mode or a non-fatiguing mode would be switched back and any possible reversible etiologies for failure would also be reviewed before the next SBT every day.

### Recording of clinical conditions and laboratory measurements

Patients’ demographics including age, gender, body mass index (BMI), and smoking and disease histories were recorded. The disease severities were assessed upon the admission to ICU by different scoring systems including acute physiology and chronic health evaluation II (APACHE II) scores, multiple organ dysfunction (Marshall) scores, sequential organ failure assessment (SOFA) scores, lung injury scores (LIS), and gas exchange, organ failure, cause, and associated disease (GOCA) scores. APACHE II is a comprehensive scoring system to identify acutely ill patients with high risk of death based on 14 parameters such as body temperature, mean arterial pressure, RR, PaO_2_, ABG, and mental status [18]. Marshall and SOFA scores are used to evaluate the organ function or rate of failure, which consists of 4 or 6 items involving cardiovascular, hepatic, coagulation, renal, respiratory, and neurological systems [19]. LIS scoring system specifically stratifies the extent of lung injury into three categories from no lung injury to severe lung injury by the infiltration area on chest X-ray, PaO_2_/FiO_2_, PEEP, and static compliance of lung [20]. Similar to LIS, GOCA score was developed for patients with ARDS to define the disease but not to predict prognosis [21].

Clinical conditions as well as the laboratory measurements at the time of ICU admission rather than extubation were recorded, including vital signs, ABG, blood cell count, blood electrolytes, myocardial, and inflammatory biomarkers. The worst (highest) level of blood glucose measured within 24 h of ICU admission was recorded. Pathogenic microorganisms were generally screened in sputum (including bronchoalveolar lavage fluid, BALF), blood, and urine by culture or chemiluminescence. We also recorded details of treatment during ICU such as mechanical ventilation settings, types and dose of analgesics and sedatives, usage of vasopressors and muscle relaxants, as well as component and amount of blood transfusions.

### Statistical analysis

Statistical analysis was performed by SPSS 21.0 [Copyright (c) SPSS Inc. 1989-2007]. Continuous data were presented as mean ± standard deviation (SD), while dichotomous data were reported as frequency and proportion. Independent Student’s *t*-test and Chi-square test or Fisher’s exact test were performed between extubation success and failure groups. Variables with two-sided *P* value of < 0.05 from the independent Student’s *t*-test and Chi-square test were individually analyzed by univariate logistic regression model, and were assessed for potential correlations and multicollinearity among individual variables by correlation matrixs and variance inflation factors (VIF). Thereafter, significant predictors with *P* value of < 0.10 were further analyzed in the multivariate logistic regression to identify the independent risk factors associated with extubation failure, and the adjusted odds ratio (OR) and 95% confidence interval (CI) were calculated. Receiver operating characteristic (ROC) curves were conducted to depict the area under the curve (AUC) for the accuracy of each independent risk factor in recognizing extubation failure, and cutoff points together with sensitivity, specificity, positive and negative predictive value (PPV and NPV), and likelihood ratio (LR) were also calculated by maximal Youden index (= sensitivity + specificity − 1).

## Results

**Figure 1:**
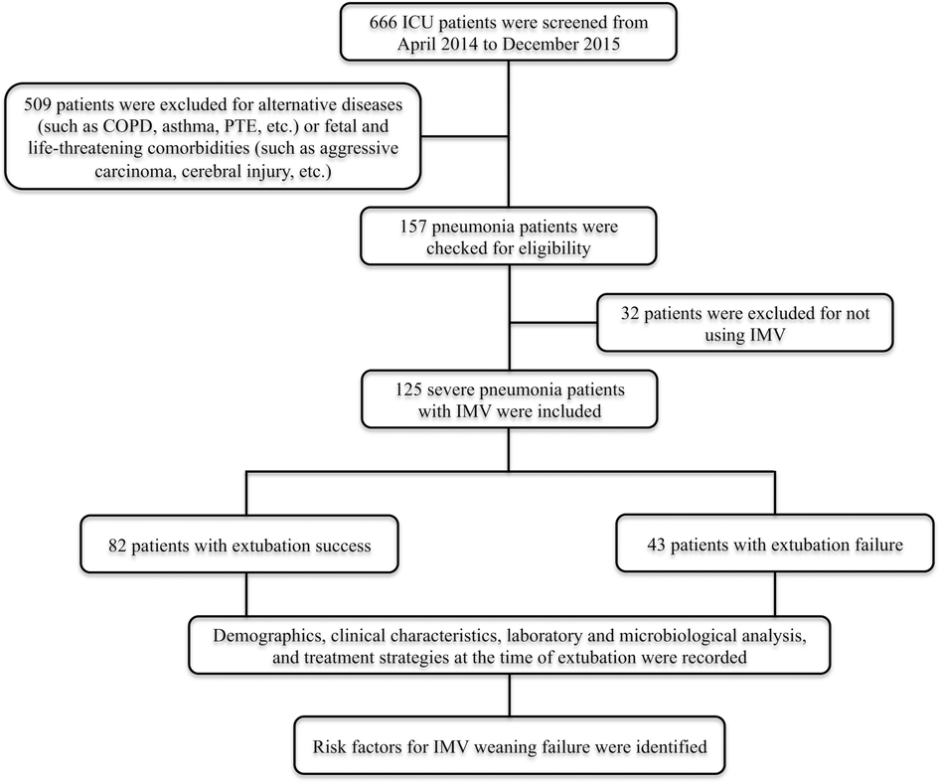
Study flow diagram Abbreviations: COPD, chronic obstructive pulmonary disease; ICU, intensive care unit; IMV, invasive mechanical ventilation; PTE, pulmonary thromboembolism.

A total of 666 patients with RICU admission were found, of which 509 patients were excluded according to the pre-defined exclusion criteria and 32 patients were excluded for not using IMV. Finally, 125 patients with severe pneumonia were included and divided into extubation success (*n* = 82) and failure (*n* = 43) groups (Figure 1). The rate of extubation failure was as high as 34.4% in patients with severe pneumonia, and the total mortality reached up to 58.4% (*n* = 73). The average days to extubation were 13.4 days.

### Baseline demographics and clinical characteristics between extubation success and failure

Tables 1 and 2 summarized patients’ baseline characteristics and clinical and laboratory measurements, respectively. Compared with patients with extubation success, patients with extubation failure were associated with significantly higher mortality (97.7% vs. 37.8%, *P* < 0.001), but no significant difference was found in ICU stay (14.42 ± 12.87 vs. 18.29 ± 15.67 days, *P* = 0.166).

**Table 1:** Baseline characteristics for patients with extubation success and failure

Characteristics	Extubation success (*n* = 82)	Extubation failure (*n* = 43)	*P*
*Demographics*			
Gender (Male, %)	57 (69.5)	24 (55.8)	0.128
Age (years)	60 ± 18	61 ± 18	0.776
BMI (kg/m^2^)	22.01 ± 2.85	21.41 ± 2.51	0.489
Smoking (%)	26 (31.7)	19 (44.2)	0.167
*Disease severity*			
APACHE II	16.30 ± 4.87	18.30 ± 4.23	0.025
Marshall	2.17 ± 2.30	2.56 ± 2.26	0.370
SOFA	5.18 ± 2.80	5.98 ± 2.75	0.132
LIS	2.19 ± 0.74	2.44 ± 0.73	0.047
GOCA	4.89 ± 1.49	5.47 ± 1.18	0.030
*Comorbidities*			
Cardiovascular diseases (%)	30 (36.6)	20 (46.5)	0.282
Diabetes (%)	17 (20.7)	12 (27.9)	0.367
Renal diseases (%)	17 (20.7)	9 (20.9)	0.979
Connective tissue diseases (%)	5 (6.1)	6 (14.0)	0.185
Gastrointestinal diseases (%)	20 (24.4)	15 (34.9)	0.215
Metabolic diseases (%)	4 (4.9)	1 (2.3)	0.659
AIDS (%)	4 (4.9)	1 (2.6)	1.000
Syphilis (%)	4 (4.9)	0 (0)	0.304
Hepatitis B (%)	4 (4.9)	2 (5.1)	1.000
*Outcomes*			
Mortality (%)	31 (37.8)	42 (97.7)	<0.001
Length of ICU stay (days)	18.29 ± 15.67	14.42 ± 12.87	0.166

Abbreviations: AIDS, acquired immune deficiency syndrome; APACHE II, acute physiology, age, chronic health evaluation; BMI, body mass index; GOCA, gas exchange, organ failure, cause, associated disease; ICU, intensive care unit; LIS, lung injury score; SD, standard deviation; SOFA, sequential organ failure assessment.

**Table 2:** Clinical characteristics and laboratory analysis at the time of extubation between patients with extubation success and failure

Parameters	Extubation success (*n* = 82)	Extubation failure (*n* = 43)	*P*
*Clinical characteristics*			
Lower extremities edema (%)	26 (31.7)	17 (39.5)	0.381
Body temperature (°C)	37.1 ± 0.9	37.1 ± 1.1	0.999
Respiratory rate (bpm)	23 ± 6	26 ± 7	0.012
Heart rate (bpm)	105 ± 24	112 ± 28	0.121
SBP (mmHg)	116 ± 22	121 ± 22	0.251
DBP (mmHg)	67 ± 15	70 ± 20	0.484
SpO_2_ (%)	96 ± 5	94 ± 8	0.162
Delirium (%)	14 (17.1)	4 (9.3)	0.240
New-onset septic shock (%)	14 (17.1)	16 (37.2)	0.012
New-onset atrial fibrillation (%)	2 (2.4)	1 (2.3)	1.000
ARDS development (%)	25 (30.5)	14 (32.6)	0.812
Imaging			
Echocardiography			
Left ventricle (cm^2^)	44.63 ± 5.02	45.33 ± 7.07	0.571
Right ventricle (cm^2^)	20.40 ± 2.71	20.59 ± 3.78	0.779
Ejection fraction (%)	64 ± 7	60 ± 15	0.070
Pericardial effusion (%)	18 (24.3)	7 (20.0)	0.616
Chest CT			
Pleural effusion (%)	55 (67.1)	29 (69.0)	0.824
Head CT			
Cerebral lacunar infarction	20 (54.1)	10 (62.5)	0.569
*Laboratory analysis*			
Blood types			
Type A (%)	16 (32.0)	5 (17.2)	0.152
Type B (%)	10 (20.0)	8 (27.6)	0.438
Type AB (%)	3 (6.0)	4 (13.8)	0.413
Type O (%)	21 (42.0)	12 (41.4)	0.957
Arterial blood gas			
pH	7.383 ± 0.069	7.350 ± 0 .105	0.039
PaO_2_/FiO_2_ (mmHg)	151.14 ± 87.92	121.14 ± 64.39	0.050
PaCO_2_ (mmHg)	43.1 ± 10.4	43.7 ± 14.7	0.779
HCO_3_^−^ (mmol/l)	25.0 ± 5.1	23.0 ± 5.1	0.055
Blood cell analysis			
Hemoglobin (g/l)	101 ± 23	102 ± 24	0.772
Hematocrit (%)	35 ± 38	31 ± 7	0.475
Platelet (10^9^/l)	164 ± 92	152 ± 98	0.505
WBC (10^9^/L)	13.00 ± 7.58	14.10 ± 9.10	0.475
Neutrophils (%)	89.2 ± 7.4	87.7 ± 12.6	0.385
*Laboratory analysis*			
Biochemical analysis			
TB (μmol/l)	10.8 ± 7.3	15.2 ± 17.7	0.048
ALT (IU/l)	56 ± 160	84 ± 230	0.434
AST (IU/l)	86 ± 223	237 ± 895	0.150
ALP (IU/l)	121 ± 90	106 ± 83	0.356
GGT (IU/l)	86 ± 125	116 ± 344	0.475
Albumin (g/l)	27.5 ± 4.8	27.8 ± 4.4	0.769
BUN (mmol/l)	10.95 ± 6.72	12.35 ± 8.24	0.307
Creatinine (μmol/l)	105.9 ± 94.2	109.1 ± 90.7	0.857
Glucose (mmol/l)	8.93 ± 3.96	10.47 ± 5.24	0.038
LDH (IU/l)	470 ± 289	793 ± 1823	0.128
Na^+^ (mmol/l)	141.3 ± 6.4	139.4 ± 6.8	0.129
K^+^ (mmol/l)	4.05 ± 0.57	4.21 ± 0.67	0.173
Cl^−^ (mmol/l)	105.7 ± 8.1	104.3 ± 8.5	0.356
Ca^2+^ (mmol/l)	1.98 ± 0.16	1.97 ± 0.21	0.690
Coagulation analysis			
PT (s)	14.1 ± 2.6	15.0 ± 5.2	0.177
INR	1.24 ± 0.22	1.32 ± 0.45	0.178
APTT (s)	37.5 ± 11.6	48.0 ± 37.0	0.021
FiB (g/l)	4.37 ± 2.00	4.53 ± 2.29	0.687
D-dimer (mg/l FEU)	9.77 ± 9.77	218.16 ± 1374.57	0.182
*Laboratory analysis*			
Myocardial biomarkers			
Myoglobin (ng/ml)	286.20 ± 557.74	530.58 ± 868.80	0.066
Troponin (ng/l)	79.04 ± 187.12	339.31 ± 1500.11	0.130
BNP (pg/ml)	3587 ± 6683	6904 ± 10049	0.034
Inflammatory biomarkers			
Procalcitonin (ng/ml)	7.22 ± 18.45	7.52 ± 16.31	0.930
CRP (ng/l)	144.79 ± 108.25	174.69 ± 140.22	0.288
Interleukin-6 (pg/ml)	598.28 ± 1230.68	872.13 ± 1432.04	0.373
Cellular immunity			
CD3 cell (%)	58.23 ± 16.28	56.33 ± 15.23	0.589
CD4 cell (%)	28.62 ± 14.75	26.67 ± 10.46	0.507
CD8 cell (%)	26.69 ± 16.41	26.55 ± 14.40	0.968
Humoral immunity			
Complement 3 (g/l)	0.8550 ± 0.2253	0.7309 ± 0.2959	0.035
Complement 4 (g/l)	0.2200 ± 0.0709	0.1957 ± 0.0958	0.193

Abbreviations: ALP, alkaline phosphatase; ALT, alanine aminotransferase; APTT, activated partial thromboplastin time; ARDS, acute respiratory distress syndrome; AST, aspartate aminotransferase; BNP, brain natriuretic peptide; BUN, blood urea nitrogen; Ca^2+^, calcium ion; Cl^−^, chloride ion; CRP, C-reactive protein; CT, computed tomography; DBP, diastolic blood pressure; FiB, fibrinogen; GGT, γ-glutamyltranspetidase; HCO^3−^, bicarbonate ion; INR, international normalized ratio; K^+^, potassium ion; LDH, lactate dehydrogenase; Na^+^, sodium ion; PaCO_2_; partial pressure of arterial carbon dioxide; PaO_2_/FiO_2_, ratio of partial pressure of arterial oxygen to fraction of inspired oxygen; PT, prothrombin time; SBP, systolic blood pressure; SpO_2_, oxygen saturation of pulse oximetry; TB, total bilirubin; WBC, white blood cell count.

In the group of extubation failure, we found significantly higher APACHE II score (18.30 ± 4.23 vs. 16.30 ± 4.87, *P* = 0.025) and blood glucose (10.47 ± 5.24 vs. 8.93 ± 3.96 mmol/l, *P* = 0.038), but significantly lower pH (7.350 ± 0.105 vs. 7.383 ± 0.069, *P* = 0.039) and plasma complement 3 level (0.7309 ± 0.2959 vs. 0.8550 ± 0.2253 g/l, *P* = 0.035) compared with the group of extubation success. Significant increase was also found in LIS score, GOCA score, RR, incidence of new-onset septic shock, total bilirubin (TB), activated partial thromboplastin time (APTT), and brain natriuretic peptide (BNP).

### Pathogenic microorganisms features between extubation success and failure

*Acinetobacter baumannii* was the most commonly detected bacterium in sputum (including BALF), blood and urine, while *Candida albicans* were the most common fungi identified in sputum (including BALF) and urine (Table 3). *Pseudomonas aeruginosa* and *Candida tropicalis* could also be easily found in sputum (including BALF) but not in blood or urine. *Herpes simplex virus* predominated the causes for severe viral pneumonia, followed by *Epstein–Barr virus*. However, we did not find any significant differences in each pathogenic microorganism from sputum (including BALF), blood and urine between the extubation success and failure.

**Table 3:** Microbiological analyses at the time of extubation between patients with extubation success and failure

Parameters	Extubation success (*n* = 82)	Extubation failure (*n* = 43)	*P*
*Sputum (BALF) culture*			
Bacteria			
*Acinetobacter baumannii* (%)	50 (61.0)	25 (64.1)	0.741
*Pseudomonas aeruginosa* (%)	16 (19.5)	5 (12.8)	0.364
*Klebsiella pneumoniae* (%)	6 (7.3)	4 (10.3)	0.725
*Burkholderia cepacia* (%)	4 (4.9)	2 (5.1)	1.000
*Stenotrophomonas maltophilia* (%)	7 (8.5)	3 (7.7)	1.000
* Enterobacter cloacae* (%)	5 (6.1)	1 (2.6)	0.663
*Serratia marcescens* (%)	4 (4.9)	0 (0)	0.304
*Escherichia coli* (%)	3 (3.7)	1 (2.6)	1.000
*Haemophilus influenzae* (%)	0 (0)	1 (2.6)	0.322
*Bacillus mirabilis* (%)	2 (2.4)	0 (0)	1.000
*Staphylococcus aureus* (%)	3 (3.7)	2 (5.1)	0.657
Fungi			
*Candida albicans* (%)	21 (33.9)	11 (31.4)	0.806
* Candida tropicalis* (%)	8 (12.9)	1 (2.9)	0.150
*Candida glabrata* (%)	5 (8.1)	0 (0)	0.156
*Candida krusei* (%)	1 (1.6)	1 (2.9)	1.000
*Candida parapsilosis* (%)	0 (0)	1 (2.9)	0.361
*Aspergillus fumigatus* (%)	2 (2.4)	2 (5.0)	0.597
*Blood culture*			
Bacteria			
* Acinetobacter baumannii* (%)	9 (13.4)	5 (13.5)	1.000
*Escherichia coli* (%)	0 (0)	1 (2.7)	0.356
* Enterococcus faecium* (%)	0 (0)	1 (2.7)	0.356
*Pseudomonas aeruginosa* (%)	1 (1.5)	0 (0)	1.000
*Urine culture*			
Bacteria			
* Escherichia coli* (%)	1 (3.4)	0 (0)	1.000
* Klebsiella pneumoniae* (%)	2 (6.9)	0 (0)	1.000
Acinetobacter baumannii (%)	2 (6.9)	0 (0)	1.000
Fungi			
*Candida tropicalis* (%)	1 (3.6)	0 (0)	1.000
* Candida glabrata* (%)	2 (7.1)	1 (10.0)	1.000
*Candida albicans* (%)	3 (10.7)	3 (30.0)	0.310
*Serum viral IgM*			
Herpes Simplex virus (%)	9 (15.5)	5 (17.2)	1.000
Rubella virus (%)	1 (1.7)	0 (0)	1.000
Cytomegalovirus (%)	0 (0)	1 (3.4)	0.330
Epstein–Barr virus (%)	4 (9.1)	2 (11.1)	1.000

Abbreviation: BALF, bronchoalveolar lavage fluid.

### Treatment and outcome characteristics between extubation success and failure

Compared with patients with extubation success, significantly higher PEEP (9 ± 4 vs. 7 ± 3 cmH_2_O, *P* = 0.021) and FiO_2_ (80 ± 22 vs. 72 ± 22%, *P* = 0.037) during IMV were needed and more midazolam (73.08 ± 55.17 vs. 48.98 ± 48.08 mg/d, *P* = 0.013) and fentanyl (0.93 ± 0.48 vs. 0.75 ± 0.45 mg/d, *P* = 0.043) were used in patients with extubation failure (Table 4). Additionally, significantly more patients used vasopressors (86.0% vs. 62.2%, *P* = 0.006) and needed transfusion of red blood cells (RBC) (44.2% vs. 28.0%, *P* = 0.040) in patients with extubation failure. The use of muscle relaxants, corticosteroids, diuretics, and insulin were similar in the two groups.

**Table 4:** Treatment strategies at the time of extubation between patients with extubation success and failure

Parameters	Extubation success (*n* = 82)	Extubation failure (*n* = 43)	*P*
IMV (%)			
Mode (A/C, %)	70 (85.4)	40 (93.0)	0.211
Frequency (bpm)	14 ± 3	14 ± 3	0.747
Tidal volume (ml)	461 ± 74	449 ± 49	0.415
Inspiratory pressure (cmH_2_O)	16 ± 4	15 ± 3	0.669
PEEP (cmH_2_O)	7 ± 3	9 ± 4	0.021
FiO_2_ (%)	72 ± 22	80 ± 22	0.037
Time to extubation (days)	13.5 ± 13.8	14.4 ± 12.9	0.739
Sequential withdrawal with NPPV (%)	31 (37.8)	0 (0)	0.000
Analgesics and sedatives			
Midazolam (mg/d)	48.98 ± 48.08	73.08 ± 55.17	0.013
Propofol (mg/d)	395.22 ± 425.31	442.19 ± 522.59	0.589
Fentanyl (mg/d)	0.75 ± 0.45	0.93 ± 0.48	0.043
Vasopressors (%)	51 (62.2)	37 (86.0)	0.006
Muscle relaxants (%)	10 (12.2)	6 (14.0)	0.780
Antibiotics			
Number of antibacterial agents	4 ± 7	4 ± 3	0.580
Number of antifungal agents	1 ± 1	1 ± 1	0.410
Number of antiviral agents	0 ± 0	0 ± 0	0.711
Venous corticosteroids (%)	54 (65.9)	31 (72.1)	0.477
Venous diuretics (%)	72 (87.8)	37 (86.0)	0.780
Insulin (%)	35 (42.7)	23 (53.5)	0.250
Blood products			
Albumin (%)	75 (91.5)	38 (88.4)	0.750
RBC (%)	23 (28.0)	19 (44.2)	0.040
Platelet (%)	9 (11.0)	3 (7.0)	0.543
Plasma (%)	18 (22.0)	13 (30.2)	0.308
Cryoprecipitate (%)	3 (3.7)	0 (0)	0.551

Abbreviations: A/C, assist/control; FiO_2_, fraction of inspired oxygen; IMV, invasive mechanical ventilation; NPPV, non-invasive positive pressure ventilation; PEEP, positive end-expiratory pressure; RBC, red blood cell.

### Predictors and risk factors for extubation failure in patients with severe pneumonia

Correlation matrix analysis showed significant correlations among the potential risk factors for extubation failure identified previously except for APACHE II, EF, blood glucose, dose of fentanyl, and percentage of patients using RBC transfusion, and variance inflation factors for these parameters showed no multicollinearity between each other (VIF ranged from 1.011 to 1.037) (data not shown). Multivariate logistic regression found APACHE II score (OR 1.141, 95% CI 1.022–1.273, *P* = 0.019), blood glucose (OR 1.122, 95% CI 1.008–1.249, *P* = 0.035), cumulative dose of fentanyl (OR 3.010, 95% CI 1.100-8.237, *P* = 0.032), and percentage of patients using RBC transfusion (OR 2.774, 95% CI 1.062–7.252, *P* = 0.037) were independent risk factors for predicting extubation failure but not EF (OR 0.965, 95%CI 0.925–1.007, *P* = 0.097) (Table 5).

**Table 5:** Univariate and multivariate logistic regression of risk factors for extubation failure in patients with severe pneumonia

Variables	Univariate logistic regression		Multivariate logistic regression	
	OR (95% CI)	*P*	OR (95% CI)	*P*
APACHEII	1.096 (1.010–1.188)	0.027	1.141 (1.022–1.273)	0.019
LIS	1.596 (0.947–2.688)	0.079		
GOCA	1.356 (1.025–1.794)	0.033		
Respiratory rate (bpm)	1.072 (1.014–1.134)	0.015		
New-onset septic shock (%)	2.878 (1.237–6.698)	0.014		
Ejection fraction (%)	0.963 (0.923–1.006)	0.092	0.965 (0.925–1.007)	0.097
pH	0.009 (0.000–0.910)	0.045		
PaO_2_/FiO_2_ (mmHg)	0.995 (0.990–1.000)	0.056		
HCO_3_^−^ (mmol/l)	0.923 (0.849–1.003)	0.059		
TB (μmol/l)	1.035 (0.995–1.076)	0.087		
Glucose (mmol/l)	1.079 (0.992–1.172)	0.076	1.122 (1.008–1.249)	0.035
APTT (s)	1.020 (1.000–1.041)	0.055		
Myoglobin (ng/ml)	1.000 (1.000–1.001)	0.078		
BNP (pg/ml)	1.000 (1.000–1.000)	0.044		
Complement 3 (g/l)	0.146 (0.023–0.912)	0.039		
PEEP (cmH_2_O)	1.132 (1.016–1.263)	0.025		
FiO_2_ (%)	1.018 (1.001–1.036)	0.039		
Sequential withdrawal with NPPV (%)	0.000 (0.000–)	0.998		
Midazolam (mg/d)	1.009 (1.002–1.017)	0.016		
Fentanyl (mg/d)	2.304 (1.011–5.247)	0.047	3.010 (1.100–8.237)	0.032
Vasopressors (%)	3.748 (1.419–9.900)	0.008		
RBC transfusion (%)	2.031 (0.939–4.390)	0.072	2.774 (1.062–7.252)	0.037

Abbreviations: APACHE II, acute physiology, age, chronic health evaluation; APTT, activated partial thromboplastin time; BNP, brain natriuretic peptide; CI, confidence interval; FiO_2_, fraction of inspired oxygen; GOCA, gas exchange, organ failure, cause, associated disease; HCO^3−^, bicarbonate ion; LIS, lung injury score; NPPV, non-invasive positive pressure ventilation; OR, odds ratio; PaO_2_/FiO_2_, ratio of partial pressure of arterial oxygen to fraction of inspired oxygen; PEEP, positive end-expiratory pressure; RBC, red blood cell; TB, total bilirubin.

**Figure 2:**
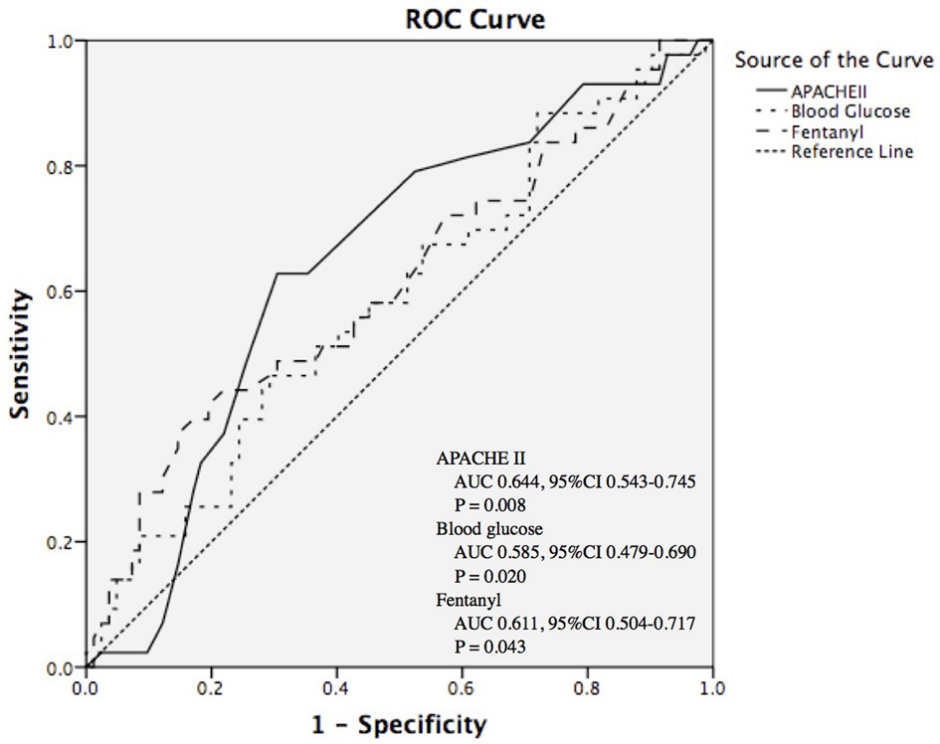
ROC curves of APACHE II, blood glucose, and fentanyl at the time of extubation in distinguishing extubation failure in patients with severe pneumonia APACHE II, acute physiology and chronic healthy evaluation II; AUC, area under the curve; CI, confidence interval; ROC, receiver operating curve.

Figure 2 depicted the ROC curves for APACHE II score, blood glucose, and dose of fentanyl, in which all these factors showed acceptable capacity in the identification of extubation failure in patients with severe pneumonia (APACHE II score: AUC 0.644, 95% CI 0.543–0.745, *P* = 0.008; blood glucose: AUC 0.585, 95% CI 0.479–0.690, *P* = 0.020; dose of fentanyl: AUC 0.611, 95% CI 0.504–0.717, *P* = 0.043). A cutoff point of 17.5 for APACHE II score, 9.87 mmol/l for blood glucose, and 1.135 mg/d for fentanyl usage resulted in a sensitivity of 62.79%, 41.86% and 37.21%, and a specificity of 69.51%, 71.95% and 85.37%, respectively, in identifying severe pneumonia patients with high risk of extubation failure, as well as a good negative predictive value (NPV: 78.08%, 70.24%, and 72.16%) (Table 6).

**Table 6:** Cut-off points of APACHE II, blood glucose, and fentanyl, and the corresponding sensitivity, specificity, PPV, NPV, and LR in distinguishing extubation failure in patients with severe pneumonia

	Cut-off point	Sensitivity (%)	Specificity (%)	PPV (%)	NPV (%)	LR+	LR-
APACHE II	17.5	62.79	69.51	51.92	78.08	2.06	0.54
Blood glucose (mmol/l)	9.87	41.86	71.95	43.90	70.24	1.49	0.81
Fentanyl (mg/d)	1.135	37.21	85.37	57.14	72.16	2.54	0.74

APACHE II, acute physiology, age, chronic health evaluation; LR, likelihood ratio; NPV, negative predictive value; PPV, positive predictive value.

## Discussion

It has been recognized that extubation failure is associated with increased mortality, longer ICU and hospital stays, and more nosocomial pneumonia [22,23]. In our study, we also demonstrated that patients with extubation failure had higher risk of death but not longer ICU stays, which mainly resulted from the death of these patients before they met the readiness of extubation. Due to the high incidence of extubation failure as well as the subsequent association of mortality, we are intrigued to explore potential factors associated with extubation failure to improve patients’ outcomes by optimizing treatment strategies and ventilator settings.

APACHE II score is the most commonly used tool to evaluate disease severity in ICU [18,24]. It has also been reported to have potential predictive values in extubation [25]. In our study, we also found that APACHE II score is an independent factor for predicting extubation failure from IMV although with a relatively low sensitivity (62.79%) and specificity (69.51%) at a cut-off point of greater than 17.5. However, we did not find such power of SOFA or Marshall score, which was not consistent with the previous finding that a high SOFA score on day 21 of IMV was associated with extubation and weaning failure in patients requiring prolonged IMV [26]. These differences might be because: (1) the different diseases for mechanical ventilation in different studies. Our study specifically focused on patients with severe pneumonia; (2) the offset effect of APACHE II score on the significance of SOFA as the items of SOFA are also included in APACHE II score. In terms of the LIS and GOCA scores, both of them were shown to be higher in patients with extubation failure, but they were not considered for multivariate logistic regression due to the significant correlations with other variables. Furthermore, LIS and GOCA are designed particularly for ARDS development, and our study did not find significant difference in ARDS development between patients with extubation failure and success.

Hyperglycemia is a common phenomenon in critically ill patients resulted from the development of hyperglycemia or insulin resistance [27,28]. It is found to be associated with increased mortality and adverse events such as postoperative infection and critical-illness polyneuropathy [29]. Studies have demonstrated that lowered blood glucose level rather than the insulin dose could bring beneficial effects [30,31], and the blood glucose control was also recognized as an independent predictor for prolonged mechanical ventilation [32,33] In our study, we also found that blood glucose of greater than 9.87 mmol/l was associated with higher risk of extubation failure, which might be due to the normal immune function maintenance, systematic inflammation reduction, endothelium and mitochondrial ultrastructure production, and skeletal muscle strength improvement after the recovery of normal glucose uptake [34,35].

Overuse and accumulation of opioid agents have been elucidated to be associated with increased adverse events, such as respiratory depression, iatrogenic withdrawal syndrome, and prolongation of time to extubation and weaning [36,37]. In our study, we also found that the use of both fentanyl and midazolam were significantly increased in patients with extubation failure, and the mean dose of fentanyl >1.135 mg/d was an independent predictor in predicting extubation failure. However, midazolam was not further assessed for potential risk factors for extubation failure due to the significant correlation in the correlation matrix although it has been found that unnecessary provision of sedatives is also associated with increased ventilation days [38]. Therefore, further studies are warranted to adjust the interdependence and interruption of sedatives such as midazolam before the conclusive consensus being made.

Previous studies found that increase of blood hemoglobin level rather than RBC transfusion treatment could improve extubation success [39–41]. Moreover, evidence also showed that hemoglobin breakdown products (heme, iron) could induce pro-inflammatory responses through various cellular signals even in the process of acute lung injury and ARDS [42], which suggests that decrease of hemoglobin level might indicate severe lung inflammation. In our study, we found similar baseline hemoglobin levels in the two patient groups, but more patients received RBC transfusion in the group with extubation failure; this implicates that the patients in extubation failure group had severer lung inflammation due to the more need for RBC transfusion, which further explained our finding that RBC transfusion was associated with extubation failure and served as an independent predictor.

Potential limitations in our study included: (1) sample size was relatively low, which might result in inaccuracy or underestimation of the power of the four independent risk factors and missing of other potential risk factors, especially the previously reported risk factor of PaO_2_/FiO_2_ for respiratory failure and outcomes, which was calculated to be at the cutoff of a statistical significance; (2) significant correlations were found among the factors identified in univariate logistic regression, which might also miss some factors of potential predictive values; and (3) the study was designed as a retrospective cohort, which disenabled us to (a) evaluate some other critical factors such as RSBI, static lung compliance, and maximal inspiratory pressure, (b) further perform subgroup analysis of different categories of extubation failure such as simple extubation, difficult extubation, and prolonged extubation, and (c) analyze the correlation between the trend of these clinical and treatment outcomes and extubation failure, which might be of greater clinical importance.

## Conclusions

In patients with severe pneumonia, APACHE II score of greater than 17.5, blood glucose of greater than 9.87 mmol/l, fentanyl usage of greater than 1.135 mg/day, and the need for RBC transfusion at the time of weaning might potentially discriminate the patients with high risk of extubation failure. Further prospective studies with large patient populations are necessitated to validate more factors, especially the ones without potential correlations, to confirm and determine the exact risk factors, as well as to establish a comprehensive scoring system or algorism for better predicting extubation failure.

## Abbreviations

ABG: arterial blood gas
APACHE II: acute physiology and chronic health evaluation II
APTT: activated partial thromboplastin time
ARDS: acute respiratory distress syndrome
AUC: area under the curve
BALF: bronchoalveolar lavage fluid
BMI: body mass index
BNP: brain natriuretic peptide
BUN: blood urea nitrogen
CI: confidential interval
COPD: chronic obstructive pulmonary disease
EF: ejection fraction
GOCA: gas exchange, organ failure, cause, associated disease
HCO_3_^−^: bicarbonate ion
IMV: invasive mechanical ventilation
LIS: lung injury score
LR: likelihood ratio
NPPV: noninvasive positive pressure ventilation
NPV: negative predictive value
OR: odds ratio
PaCO_2_: partial pressure of arterial carbon dioxide
PaO_2_/FiO_2_: ratio of partial pressure of arterial oxygen to fraction of inspired oxygen
PEEP: positive end-expiratory pressure
PPV: positive predictive value
RBC: red blood cell
RICU: respiratory and infectious intensive care unit
ROC: receiver operating characteristic
RR: respiratory rate
RSBI: rapid shallow breathing index
SaO_2_: arterial oxygen saturation
SBT: spontaneous breathing trial
SD: standard deviation
SOFA: sequential organ failure assessment
SpO_2_: oxygen saturation of pulse oximetry
TB: total bilirubin
VIF: variance inflation factors
*V*_T_: tidal volume
